# Claudin5a is required for proper inflation of Kupffer's vesicle lumen and organ laterality

**DOI:** 10.1371/journal.pone.0182047

**Published:** 2017-08-03

**Authors:** Jeong-gyun Kim, Sung-Jin Bae, Hye Shin Lee, Ji-Hyeon Park, Kyu-Won Kim

**Affiliations:** 1 Department of Molecular Medicine and Biopharmaceutical Science, Graduate School of Convergence Science and Technology, Seoul National University, Seoul, Korea; 2 SNU-Harvard NeuroVascular Protection Research Center, College of Pharmacy and the Research Institute of Pharmaceutical Sciences, Seoul National University, Seoul, Korea; 3 Crop Biotechnology Institute, GreenBio Science and Technology, Seoul National University, Pyeongchang, Korea; Istituto per la Ricerca e la Cura del Cancro di Candiolo, ITALY

## Abstract

Left-right asymmetric organ development is critical to establish a proper body plan of vertebrates. In zebrafish, the Kupffer’s vesicle (KV) is a fluid-filled sac which controls asymmetric organ development, and a properly inflated KV lumen by means of fluid influx is a prerequisite for the asymmetric signal transmission. However, little is known about the components that support the paracellular tightness between the KV luminal epithelial cells to sustain hydrostatic pressure during KV lumen expansion. Here, we identified that the *claudin5a* (*cldn5a*) is highly expressed at the apical surface of KV epithelial cells and tightly seals the KV lumen. Downregulation of *cldn5a* in zebrafish showed a failure in organ laterality that resulted from malformed KV. In addition, accelerated fluid influx into KV by combined treatment of forskolin and 3-isobutyl-1-methylxanthine failed to expand the partially-formed KV lumen in *cldn5a* morphants. However, malformed KV lumen and defective heart laterality in *cldn5a* morphants were significantly rescued by exogenous *cldn5a* mRNA, suggesting that the tightness between the luminal epithelial cells is important for KV lumen formation. Taken together, these findings suggest that *cldn5a* is required for KV lumen inflation and left-right asymmetric organ development.

## Introduction

The internal organs, such as the heart, pancreas, and liver, are asymmetrically located along the midline of the body, even though the external body plane of vertebrates seems symmetric. However, about 1.1 per 10000 new born babies every year show failures of left-right asymmetric organ development, *situs inversus totalis* or *situs ambiguous* [1, 2]. In addition, one fourth of patients with laterality defects also suffer from primary ciliary dyskinesia, a dysfunction of the cilia in the respiratory tract, sperm cells, or fallopian tube [3, 4]. This high correlation between ciliary function and laterality defects enabled the discovery of the left-right organizer (LRO), a transient ciliary organ that controls left-right asymmetric development in vertebrates. The node in mice, gastrocoel roof plate in *Xenopus*, Hensen’s node in chick, and Kupffer’s vesicle (KV) in zebrafish were identified as LROs; they show similar, but distinct shapes and sizes, ranging from flat to indented and dome-shaped to spherical, depending on the model organism [5, 6].

KV, discovered in 1868, is a distinctive fluid-filled epithelial sac and exists transiently during the early segmentation period at the posterior end of the notochord in zebrafish [7, 8]. KV is derived from a cluster of dorsal forerunner cells (DFCs), which is maintained by cadherin-based adherens junction, and actively migrates towards the vegetal pole showing filopodia and lamellipodia and passively adheres to the overlying surface enveloping layer (EVL) until planar cell polarity signaling reconstructs the cell structure [7–12]. In addition, DFCs are prevented from intermingling with neighboring non-DFCs by Eph/ephrin signaling [13]. Then, migrated DFCs become polarized to form a rosette-like structure and the lumen forms at the apical point of the rosette structure [9]. The KV lumen is expanded by fluid influx and ciliogenesis occurs simultaneously with lumen expansion inside KV [7, 14–18]. In addition, together with *rock2b*-mediated cytoskeletal rearrangement, the notochord affects regional cell shape changes by the accumulation of extracellular matrix [19–21]. Thus, cilia are more abundantly distributed on the anterior-dorsal region. Motile monocilia generate fluid flow in the counterclockwise direction and promote Ca^2+^ elevation at the left side of the KV via the membrane-targeted calmodulin-dependent protein kinase (CaMK)-II, Pkd2, and Ryr3 [22–25]. Then, *dand5*, which is a member of the Cerberus/Dan family and antagonizes Spaw function, is expressed bilaterally around the KV lumen at 6 somite stage (ss) and later becomes predominant on the right side [26, 27]. Thus, Spaw, a *nodal*-related protein, is transmitted through the left lateral plate mesoderm, and midline molecular barriers restrict Spaw expression to the left side [28–31]. Finally, *nodal* signaling induces left-right asymmetric organ development from organ primordia [32–34].

As a fluid-filled organ, the properly inflated KV lumen is important for robust left-right patterning. There is a minimum lumen size threshold for a functional KV and an over-inflated KV lumen, induced by reinforced activation of the Cftr channel, also shows disrupted regional cell shape changes of KV epithelial cells and defective left-right asymmetry [19, 35]. The KV lumen is expanded by fluid influx through the Cftr channel, which is regulated by intracellular cAMP levels and ion gradients [14, 15, 23]. In addition, polycystin-2-dependent intracellular Ca^2+^ maintains the basal level of cAMP and prevents the over-inflation of KV [18]. During KV lumen expansion, epithelial cells lining the KV lumen should adhere tightly to sustain hydrostatic pressure of the fluid influx during KV lumen expansion. Zonula occludens-1 (ZO-1), the cytoplasmic scaffold protein that anchors tight junction proteins to the cytoskeleton, is expressed at the apical point of KV epithelial cells. However, the functional tight junction protein, constituting and tightly sealing the intercellular spaces of KV epithelial cells during apical clustering and lumen inflation, remains unknown [9, 14].

Claudins (Cldns) are key integral proteins with a critical role in supporting tight junctions between epithelial or endothelial cells, and twenty-four members of the claudin family have been identified in mammals [36]. Cldn5 is the dominant type in the brain endothelial cells, which maintains the blood-brain barrier permeability; the loss of Cldn5 in mice results in the size-selective loosening of the blood-brain barrier [37–41]. In zebrafish, two types of *cldn5*, i.e., *cldn5a* and *cldn5b*, have been identified, and their roles in the central nervous system and vascular development, respectively, have been studied. [42, 43].

In this study, we verified *cldn5a* expression in KV, supposing that the expanding KV lumen must be sealed tightly. Then, we elucidated the roles of *cldn5a* in left-right asymmetric development by *cldn5a* downregulation in KV lineage cells. In addition, we treated pharmacological reagents to validate whether the paracellular tightness between KV cells was sustained in the absence of *cldn5a*. Taken together, we demonstrated novel *cldn5a* expression in the KV and its role in KV lumen expansion and left-right asymmetric development.

## Materials and methods

### Ethics statement

All zebrafish work was carried out in accordance with protocols approved by the Institutional Animal Care and Use Committees of Seoul National University.

### Zebrafish

Tuebingen wild-type zebrafish was purchased from the Zebrafish International Resource Center (Oregon, USA), and *Tg(sox17*:*egfp)*^*s870*^ zebrafish embryos were obtained from Zebrafish International Resource Center through Zebrafish Organogenesis Mutant Bank (Daegu, Korea) [44]. *Tg(sox17*:*egfp)*^*s870*^ embryos were used for all immunofluorescence experiment except S1 Fig.

### Real-time polymerase chain reaction (qPCR)

Target-specific primer pair, cDNA, ROX dye and SyBr Master Mix were prepared for reaction. The reaction conditions were as follows: initial denaturation at 95°C for 10 min, 40 cycles of denaturation at 95°C for 15 sec, annealing at 55°C for 30 sec and elongation at 72°C for 30 sec. PCR reaction was performed using StepOnePlus real-time PCR system (Applied Biosystems). Primer sequences for PCR amplification are as below: *dand5* forward (5’-GCC GTT AGT CAT GTG CCG TT-3’) and reverse (5’-CTA TGG GTC AGG ATT GCG GG-3’), *eef1a1l1* forward (5’-CTG GAG GCC AGC TCA AAC AT-3’) and reverse (5’-ATC AAG AAG AGT AGT ACC GCT AGC ATT AC-3’).

### Whole-mount RNA *in situ* hybridization

Specific regions for *cmlc1*, *spaw*, *dand5*, *cldn5a* and *cldn5b* were cloned into pGem-T easy vector (Promega). Constructed vectors were linearized and transcribed using DIG-labeling mix, SP6, T7 RNA polymerase (Roche). The embryos were fixed with 4% paraformaldehyde (PFA) for overnight at 4°C and dehydrated with methanol at –20°C for long storage. Then, the embryos were treated with proteinase K for 1 to 10 min at room temperature depending on the developmental stages. After the proteinase K treatment, the embryos were transferred to the pre-hybridization solution (50% formamide, 5X Sodium Saline Citrate [SSC], 5 mg/mL yeast tRNA, 50 μg/mL heparin, 0.1% Tween 20) and incubated for 3 hours at 65°C. Pre-hybridization solution was replaced with the mixture solution which contains digoxigenin (DIG)-labeled RNA probe and the embryos were incubated at 65°C overnight. A series of washing steps (50% formamide/2X SSC, 2X SSC, 0.2X SSC, phosphate-buffered saline-Tween 20 [PBST]) were performed and the embryos were treated with anti-DIG-alkaline phosphatase fragment diluted 1: 1000 in blocking solution (0.5% Roche blocking reagent, 5% goat serum in PBST). After washing 15 times with PBST for 15 min, the embryos were immersed in staining solution (100 mM Tris-HCl pH 9.5, 50 mM MgCl_2_, 100 mM NaCl, 0.1% Tween 20) three times for 10 min. Then, the embryos were colorized using nitrobuleterazolium/5-bromo, 4-chloro, 3-indolylphosphate to produce insoluble purple precipitates. Stained embryos were fixed with 4% PFA for 20 min, and dehydrated with methanol for 10 min. Then, embryos were mounted in glycerol and photographed with AxioCam ICC-1 camera on Zeiss Stemi 2000C.

To analyze *dand5* expression, U-shaped and symmetric expression was categorized as normal in 6 ss embryos. In 8 ss embryos, right-sided robust expression was classified as normal.

Primer sequences to construct *cmlc1*, *spaw*, *dand5*, *cldn5a* and *cldn5b* vector are as below: *cmlc1* forward (5’-CCC AGC CTT TTC CCA TCA GCA TCA TG-3’) and reverse (5’-GCG TCG GCT CAC CCG GAG AG-3’), *spaw* forward (5’-TGC AGC CGG TCA TAG CGT GC-3’) and reverse (5’-AGA AAA CGC CGG CAG CCG AA-3’), *dand5* forward (5’-CGC GTT TCC CGC GTT CTT GG-3’) and reverse (5’-TTG TCA CGC GCC CTG GTT GA-3’), *cldn5a* forward (5’-CAA GAA TTC CAT GGC CTC CGC GGC TTT GGA-3’) and reverse (5’-CAA TCT AGA TCA CAC GTA ATT CCT CTT GT-3’), *cldn5b* forward (5’-CAA GAA TTC CAT GGC AAA TAT GAT TTC TGC-3’) and reverse (5’-CAA TCT AGA TCA GAC GTA GTT TCG TTT AT-3’).

### Whole mount immunofluorescence

The embryos were fixed with 4% PFA for overnight at 4°C and dehydrated with methanol and stored at –20°C. Then the embryos were treated with collagenase I for 10 to 45 min at room temperature depending on the developmental stages. After the collagenase I treatment, the embryos were transferred to the blocking solution (5% bovine serum albumin, 10% goat serum in PBST) and incubated for 3 hours at room temperature. Blocking solution was replaced with primary antibody containing solution and the embryos were incubated at 4°C overnight. Primary antibodies are anti-Cldn5 (1:50, 35–2500, Invitrogen), anti-ZO-1 (1:50, 339100, Invitrogen) and anti-acetylated tubulin (1:200, T6793, Sigma-Aldrich). A series of washing steps (1% dimethyl sulfoxide [DMSO], 0.5% Triton X-100 in PBST) were performed and the embryos were treated with AF-405, 488, 546 labeled anti-mouse or rabbit IgG in blocking solution for overnight at 4°C. Then, the embryos were washed with PBDTT (1% DMSO, 0.5% Triton X-100 in PBST) for 10 times. Stained embryos were mounted in glycerol and images were obtained by Zeiss LSM700 confocal microscope with ZEN software.

### Morpholino microinjection

1 ng of *cldn5a* translation-blocking morpholino^1^ (MO^1^), 2 ng of *cldn5a* translation-blocking MO^2^ which targets 5’-UTR and ATG region, respectively, and 2 ng of standard control MO were injected into the yolk at one-cell stage. For specific knockdown at DFCs, MOs were injected into the yolk at 128 to 512-cell stage (referred to as DFC MO). For yolk-specific injection, MOs were injected into the yolk at sphere to dome stage (referred to as Yolk MO). MOs were purchased from Gene Tools, LLC. Sequences of each MO are as follows: *cldn5a* MO^1^ (5’- GTA CTA AAA GGA GTT TAG AAG TTT G-3’), *cldn5a* MO^2^ (5’-AGG CCA TCG CTT TCT TTT CCC ACT C-3’), and standard control MO (5’-CCT CTT ACC TCA GTT ACA ATT TAT A-3’).

### Pharmacological treatment

Forskolin and 3-isobutyl-1-methylxanthine (IBMX) were purchased from Sigma-Aldrich (F6886 and I7018, respectively). Each reagent was primarily stocked for 10 mM and 100 mM in DMSO, respectively. Embryos were treated with the mixture of 10 μM forskolin and 40 μM IBMX from 90% epiboly to 6 ss.

### *In vitro* mRNA transcription

The coding sequence of *cldn5a* and *cldn5b* were amplified by PCR with reverse-transcribed zebrafish cDNA as a template and cloned into pCS2+ vector with N-terminal tagged mCherry, respectively. Primer pairs for *cldn5a* forward (5’-CAA GAA TTC CAT GGC CTC CGC GGC TTT GGA-3’) and reverse (5’-CAA TCT AGA TCA CAC GTA ATT CCT CTT GT-3’), and *cldn5b* forward (5’-CAA GAA TTC CAT GGC AAA TAT GAT TTC TGC-3’) and reverse (5’-CAA TCT AGA TCA GAC GTA GTT TCG TTT AT-3’) were used. Immunoreactivity of mCherry-Cldn5a and Cldn5b to anti-Cldn5 antibody were validated by immunostaining (S1 Fig). Each linearized construct was injected into embryos, and fixed at 6 hours-post fertilization (hpf), before endogenous Cldn5 to be expressed. 5’-capped *mCherry* and *cldn5a* mRNAs were generated using mMessage mMachine SP6 transcription kit (Ambion, AM1340) and poly(A)-tailing reaction was performed using 5X E-PAP Buffer, 25 mM MnCl_2_ and ATP solution in mMessage mMachine T7 ultra kit (Ambion, AM1345). 80–120 pg of *mCherry* or *mCherry*-*cldn5a* mRNA was co-injected with MO^1^.

### Statistical analysis

Measurements of KV lumen area and the number and size of cilia were performed using ZEN application (Zeiss). KV lumen area was obtained by measuring the gross area in maximum intensity projection image using Closed Beizer tool of ZEN software. KV cilia size was obtained by measuring the length in maximum intensity projection image using Line tool of ZEN software. Comparisons between control and *cldn5a* morphants were analyzed in Prism 5 (GraphPad Software, Inc.), and *P*-values were calculated by applying the unpaired two-tailed Student’s *t* test.

## Results

### *Cldn5a* is expressed in KV

**Fig 1:**
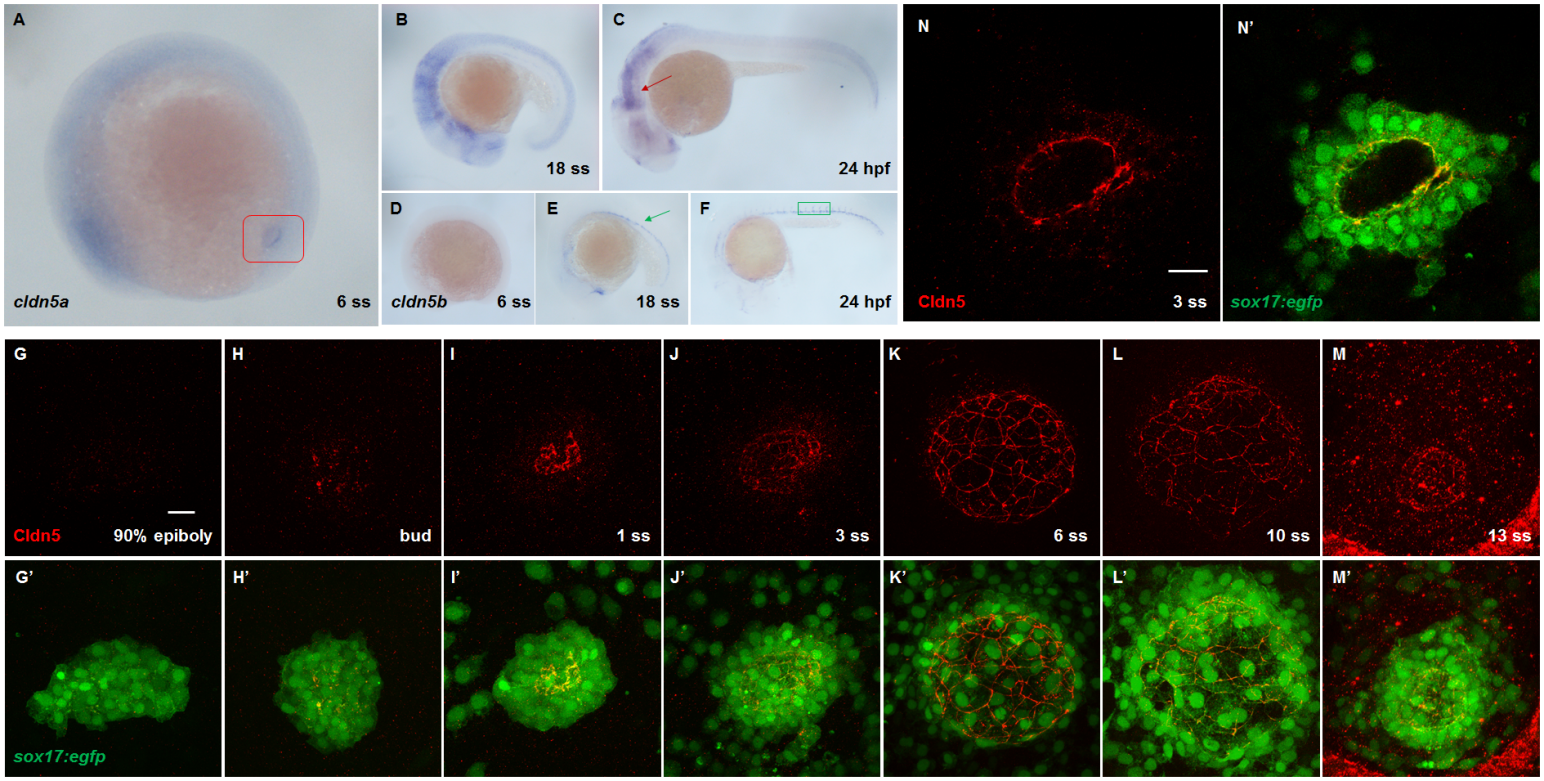
Expression patterns of *cldn5a* and *cldn5b*. **(A—C)** *In situ* hybridization of *cldn5a*. Specific expression of *cldn5a* in KV at 6 ss, marked by a red rectangle (A). Expression of *cldn5a* in neuroepithelial cells at 18 ss (B) and 24 hpf (C), marked by a red arrow. **(D—F)** *In situ* hybridization of *cldn5b*. No obvious expression of *cldn5b* at 6 ss (D). Expression of *cldn5b* in dorsal aorta (DA) and intersegmental vessels (ISV) at 18 ss (E) and 24 hpf (F). DA and ISV are marked by a green arrow and rectangle, respectively. **(G—M)** Maximum intensity projection images of Cldn5 (red) and *sox17*:*egfp*-positive KV cells (green) in 90% epiboly to 13 ss embryos. **(N)** Single plane image of Cldn5 (red) and *sox17*:*egfp*-positive KV cells (green) in 3 ss embryos. Scale bar: 20 μm.

The spatiotemporal expression patterns of *cldn5a* and *cldn5b* were examined in zebrafish by *in situ* hybridization. *cldn5a* was highly expressed within the developing cerebrum, cerebellum, and rhombomeres (red arrow in Fig 1C), while *cldn5b* was highly expressed in the dorsal aorta and intersegmental vessels as previously reported (green arrow in Fig 1E and green rectangle in Fig 1F, respectively) [42, 43]. Interestingly, it was found that *cldn5a* is expressed in KV at the 6 ss (red rectangle in Fig 1A). To ascertain the serial expression of *cldn5a* in KV development, embryos were analyzed by immunofluorescence staining with a specific antibody in transgenic zebrafish *Tg(sox17*:*egfp*)^*s870*^ expressing green fluorescence proteins in KV lineage cells [44]. Though the anti-mammal Cldn5 antibody captured both Cldn5a and Cldn5b (S1 Fig), the expression of Cldn5 in KV was efficiently suppressed by two types of *cldn5a* translation-blocking MO (S2A–S2C Fig). Cldn5 expression was not significant in *sox17*:*egfp*-positive cells at 90% epiboly, when the DFCs migrate collectively (Fig 1G). From the bud to 13 ss, however, Cldn5 was stably expressed in KV cells, while DFCs undergo apical clustering, lumen formation, lumen expansion and KV degeneration (Fig 1H–1M). Moreover, Cldn5 was highly expressed at the luminal surface of the developing KV (Fig 1N). In addition, a qPCR analysis indicated that *cldn5a* mRNA expression increased gradually as KV progenitor cells collectively migrated and underwent KV formation (S3 Fig). These data demonstrate that *cldn5a* is highly expressed at the luminal surface of KV during KV lumen formation and expansion.

### Heart laterality is disrupted in *cldn5a* morphants

**Fig 2:**
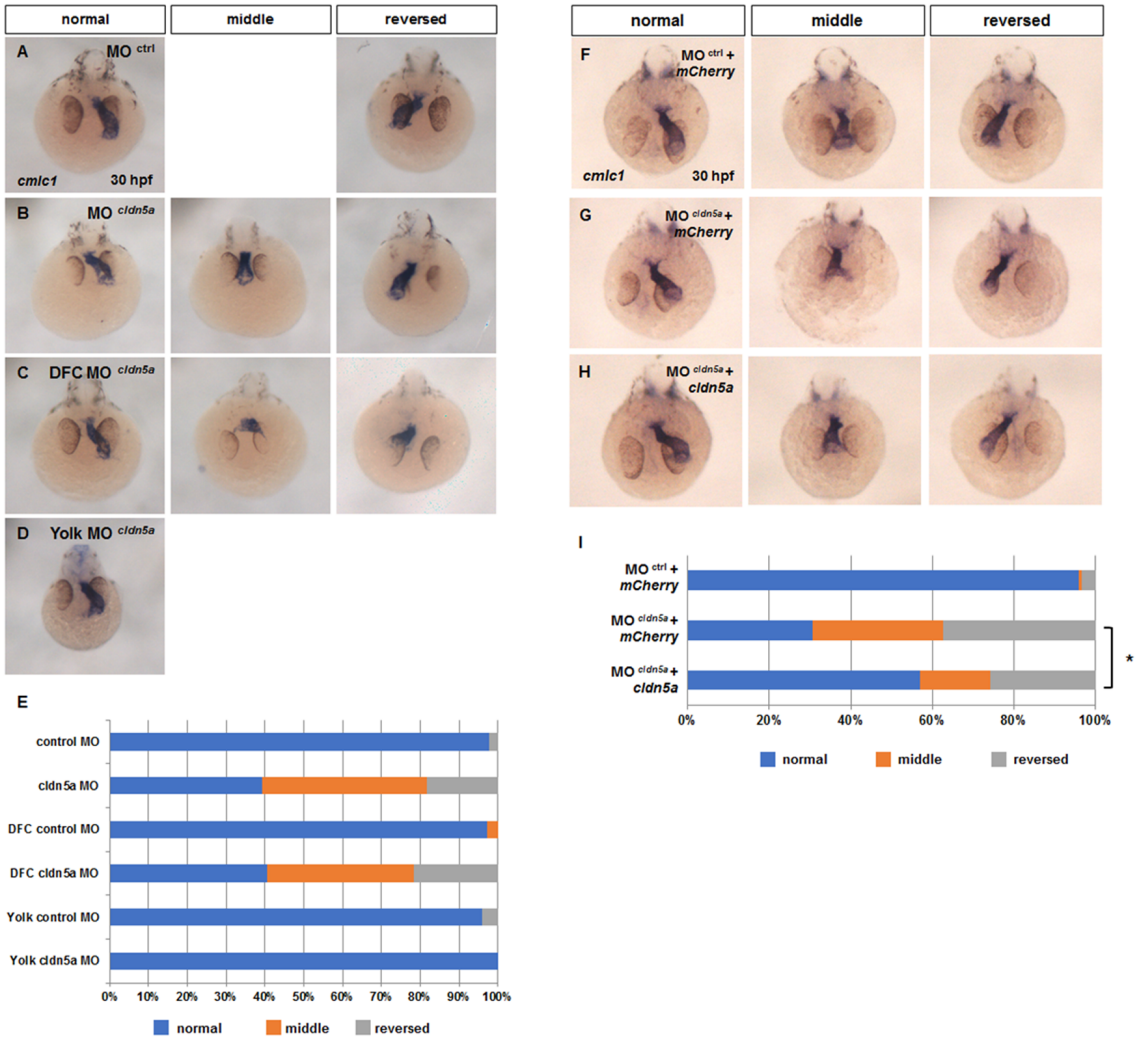
Laterality of heart was disrupted in *cldn5a* morphants. **(A—H)** Visualization of a heart by *in situ* hybridization of *cmlc1* in 30 hpf embryos. Representative images of control morphants (A), *cldn5a* morphants (B), DFC *cldn5a* morphants (C), and yolk *cldn5a* morphants (D). **(E)** Stacked bar graph (blue; normal, orange; middle, grey; reversed, control morphants; n = 45, *cldn5a* morphants; n = 33, DFC control morphants; n = 71, DFC *cldn5a* morphants; n = 37, yolk control morphants; n = 25, yolk *cldn5a* morphants; n = 31). Representative images of control morphants with *mCherry* (F), *cldn5a* morphants with *mCherry* (G), *cldn5a* morphants with *mCherry-cldn5a* (H). **(I)** Stacked bar graph (blue; normal, orange; middle, grey; reversed, control morphants with *mCherry*; n = 132, *cldn5a* morphants with *mCherry*; n = 111, *cldn5a* morphants with *mCherry-cldn5a*; n = 115). * depicts *p* < 0.05.

Specific expression of *cldn5a* in KV lineage cells suggests that *cldn5a* might influence asymmetric organ development in zebrafish. Thus, the status of the heart, a representative asymmetric organ, was investigated by *in situ* hybridization of *cmlc1* in *cldn5a*-downregulated embryos. Two types of *cldn5a* MO efficiently blocked Cldn5 expression in KV (S2A–S2C Fig) and disrupted heart laterality (S4A–S4D Fig), without severe gross morphological defects (S4E–S4G Fig). Thus, we investigated the role of *cldn5a* using 5’-UTR-targeting MO^1^, one of the two MO types. Compared with control morphants, *cldn5a* morphants exhibited significantly higher rates of the middle (42%) and reversed (18%) form of a heart (Fig 2A, 2B and 2E). To verify that the aberrant heart formation in *cldn5a* morphants was caused by *cldn5a* deficiency in KV lineage cells, we injected MO into the yolk at the 128 to 512-cell stage (DFC MO) for the exclusive reduction of *cldn5a* in DFCs (S2D–S2F Fig) and at the sphere to dome stage (Yolk MO) to confirm the effect of MO in the yolk and yolk syncytial layer. DFC *cldn5a* morphants showed a high rate of disrupted heart laterality (59%), whereas DFC-specific control, yolk-specific control, and yolk-specific *cldn5a* morphants showed a very low rate of heart laterality defects (Fig 2C–2E). Finally, *mCherry* or *mCherry-cldn5a* mRNA with *cldn5a* MO was injected to rescue heart laterality defects in *cldn5a* morphants. In *cldn5a* morphants with *mCherry*, 31% of embryos showed a left-sided normal heart; however, 57% of *cldn5a* morphants with *mCherry*-*cldn5a* exhibited a normal heart status (Fig 2F–2I). Thus, these data demonstrated that *cldn5a*, which is expressed in KV lineage cells, is functionally required for the left-right asymmetric development of the heart.

### Signal transfer from KV to organ primordia is disrupted in DFC *cldn5a* morphants

**Fig 3:**
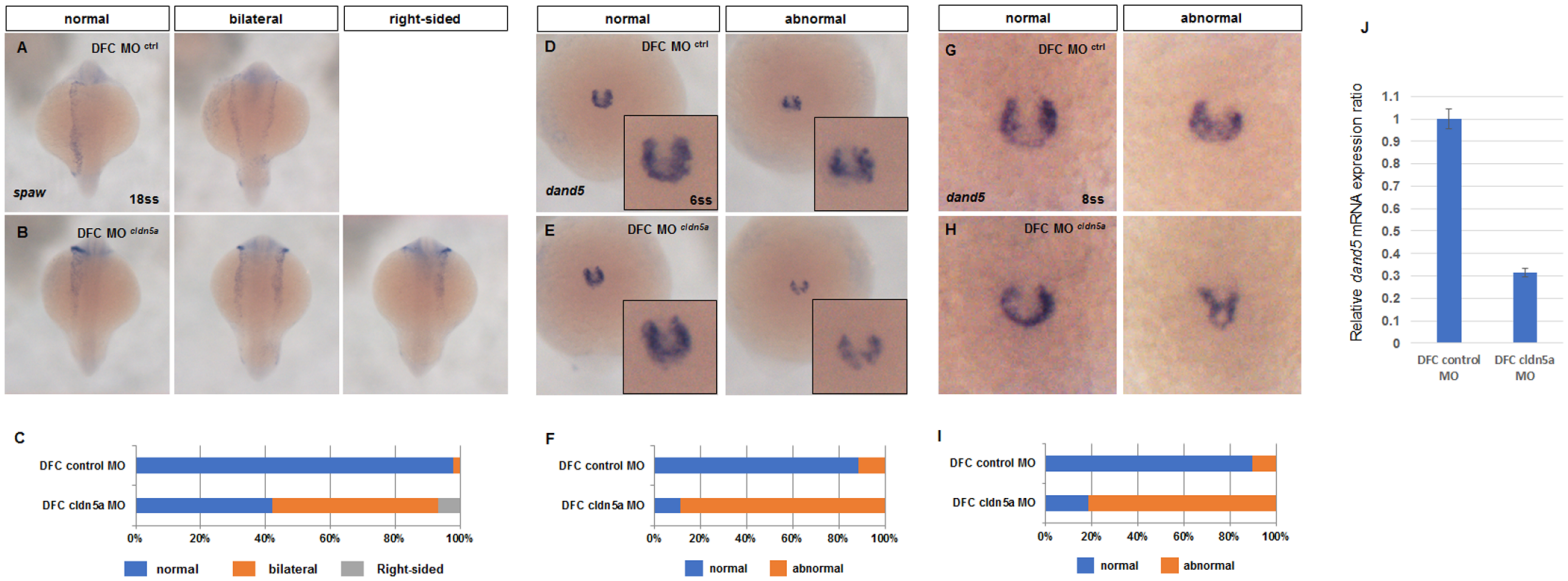
Expressions of *spaw* and *dand5* were disrupted in DFC *cldn5a* morphants. **(A—B)** Visualization of *spaw* by *in situ* hybridization in 18 ss embryos. Representative images of DFC control morphants (A) and DFC *cldn5a* morphants (B). **(C)** Stacked bar graph (blue; normal, orange; bilateral, grey; right-sided, DFC control morphants; n = 48, DFC *cldn5a* morphants; n = 45). **(D—E)** Visualization of *dand5* by *in situ* hybridization in 6 ss embryos. Representative images of DFC control morphants (D) and DFC *cldn5a* morphants (E). **(F)** Stacked bar graph (blue; normal, orange; abnormal, DFC control morphants; n = 26, DFC *cldn5a* morphants; n = 36). **(G—H)** Visualization of *dand5* by *in situ* hybridization in 8 ss embryos. Representative images of DFC control morphants (G) and DFC *cldn5a* morphants (E). **(I)** Stacked bar graph (blue; normal, orange; abnormal, DFC control morphants; n = 76, DFC *cldn5a* morphants; n = 82). **(J)** Relative *dand5* mRNA expression in DFC *cldn5a* morphants versus DFC control morphants at 8 ss. Error bars indicate s.e.m.

To validate how defective heart laterality in *cldn5a* morphants resulted from the specific downregulation of *cldn5a* in KV lineage cells, we performed *in situ* hybridization of *spaw*, the *nodal*-related gene, which propagates through the left lateral plate mesoderm (LPM) from KV and determines the laterality of organs [28, 29]. DFC *cldn5a* morphants showed bilateral (51%) and right-sided (7%) *spaw* expression, while only 2% of the DFC control morphants showed bilateral *spaw* expression (Fig 3A–3C). Next, the expression of *dand5*, which is expressed around the KV and acts as a molecular barrier of *spaw*, was investigated [26, 27]. *dand5* is expressed bilaterally around KV at 6 ss and is subsequently predominant on the right [27]. Expression patterns of *dand5* at 6 ss in DFC control morphants were mostly normal with a horseshoe shape; however, most DFC *cldn5a* morphants showed abnormal (89%) *dand5* expression (Fig 3D–3F). In addition, *dand5* expression was not predominant on the right side of KV in most of DFC *cldn5a* morphants (Fig 3G–3I). Furthermore, quantitative analysis of *dand5* revealed the significant reduction in DFC *cldn5a* morphants (0.313 ± 0.018, Fig 3J). Thus, these data showed that the disrupted heart laterality is correlated with aberrant expression of asymmetric signals.

### Ciliogenesis in DFC *cldn5a* morphants

**Fig 4:**
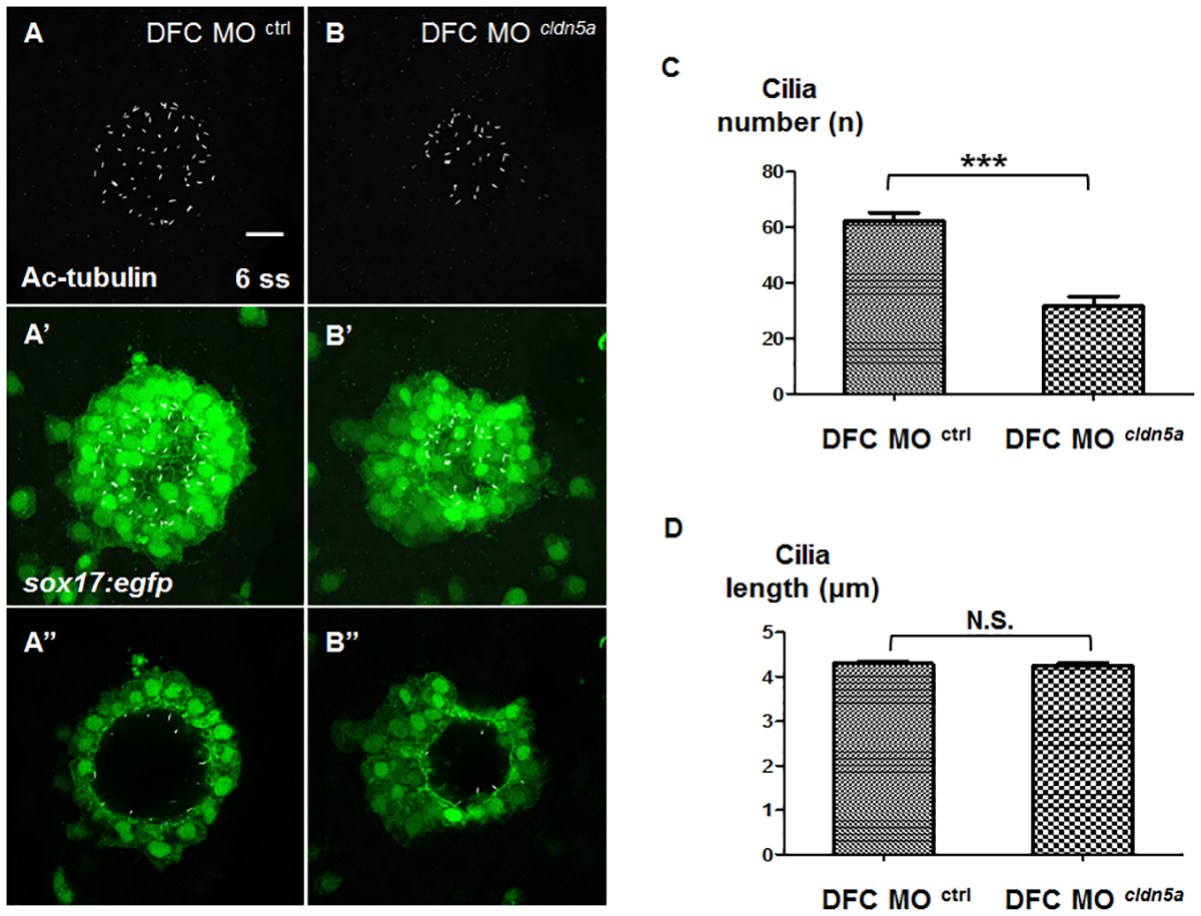
Decreased number of cilia in partially formed KV lumen. **(A—A’, B—B’)** Maximum intensity projection images of acetylated tubulin (grey) and *sox17*:*egfp*-positive KV cells (green) in 6 ss embryos. Representative images of DFC control morphants (A), and DFC *cldn5a* morphants (B). **(A”, B”)** Single plane images of acetylated tubulin (grey) and *sox17*:*egfp*-positive KV cells (green) in 6 ss embryos. **(C)** Statistical column bar graph of cilia number (DFC control morphants; n = 25, DFC *cldn5a* morphants; n = 30) **(D)** Statistical column bar graph of cilia length (DFC control morphants; n = 314, DFC *cldn5a* morphants; n = 128). *** depicts *p* < 0.001, N.S. (not significant) depicts *p* > 0.05. Error bars indicate s.e.m. Scale bar: 20 μm.

Ciliogenesis is an important event in asymmetric signal transmission. Motile cilia generate unidirectional fluid flow, and the leftward signal is activated by intracellular Ca^2+^ release [22–25]. In addition, it was reported that cilia-driven fluid flow is important for *dand5* expression [27, 45]. Thus, we investigated the status of cilia in DFC *cldn5a* morphants by immunostaining of acetylated tubulin. The numbers of cilia were 62.12 ± 3.18 and 31.57 ± 3.62 per embryo in DFC control and *cldn5a* morphants, respectively (Fig 4A–4C). Although the number of cilia decreased in DFC *cldn5a* morphants, the average length of cilia in DFC *cldn5a* morphants (4.30 ± 0.050 μm) was comparable to that of the DFC control morphants (4.24 ± 0.075 μm) (Fig 4D). Since a common feature of the downregulation of ciliogenesis factors in the KV was a shortened cilia length as well as a decreased cilia number [17, 46–49], it is suspected that *cldn5a* might not be directly involved in ciliogenesis. In this regard, we further identified the reduction of *sox17*:*egfp*-positive KV lineage cells in DFC *cldn5a* morphants from the bud to 10 ss (S5D–S5N Fig). The number of *sox17*:*egfp*-positive cells in DFC *cldn5a* morphants at the bud stage was comparable to that of DFC control morphants. In addition, though the difference of the number of *sox17*:*egfp*-positive KV cells between the DFC control and *cldn5a* morphants started to increase at 3 ss, it did not exceed 25% until 10 ss. This was still less than the reduced rate of cilia in DFC *cldn5a* morphants (49%) (Fig 4C). Instead, single plain images showed that some *sox17*:*egfp*-positive cells did not participate in KV lumen formation and that the cilia were only localized near the small KV lumen in DFC *cldn5a* morphants (Fig 4A” and 4B”). Thus, these data suggest that the decreased number of cilia within smaller KV lumen might influence the expression and asymmetry of *dand5*.

### *Cldn5a* is required for proper inflation of KV lumen

**Fig 5:**
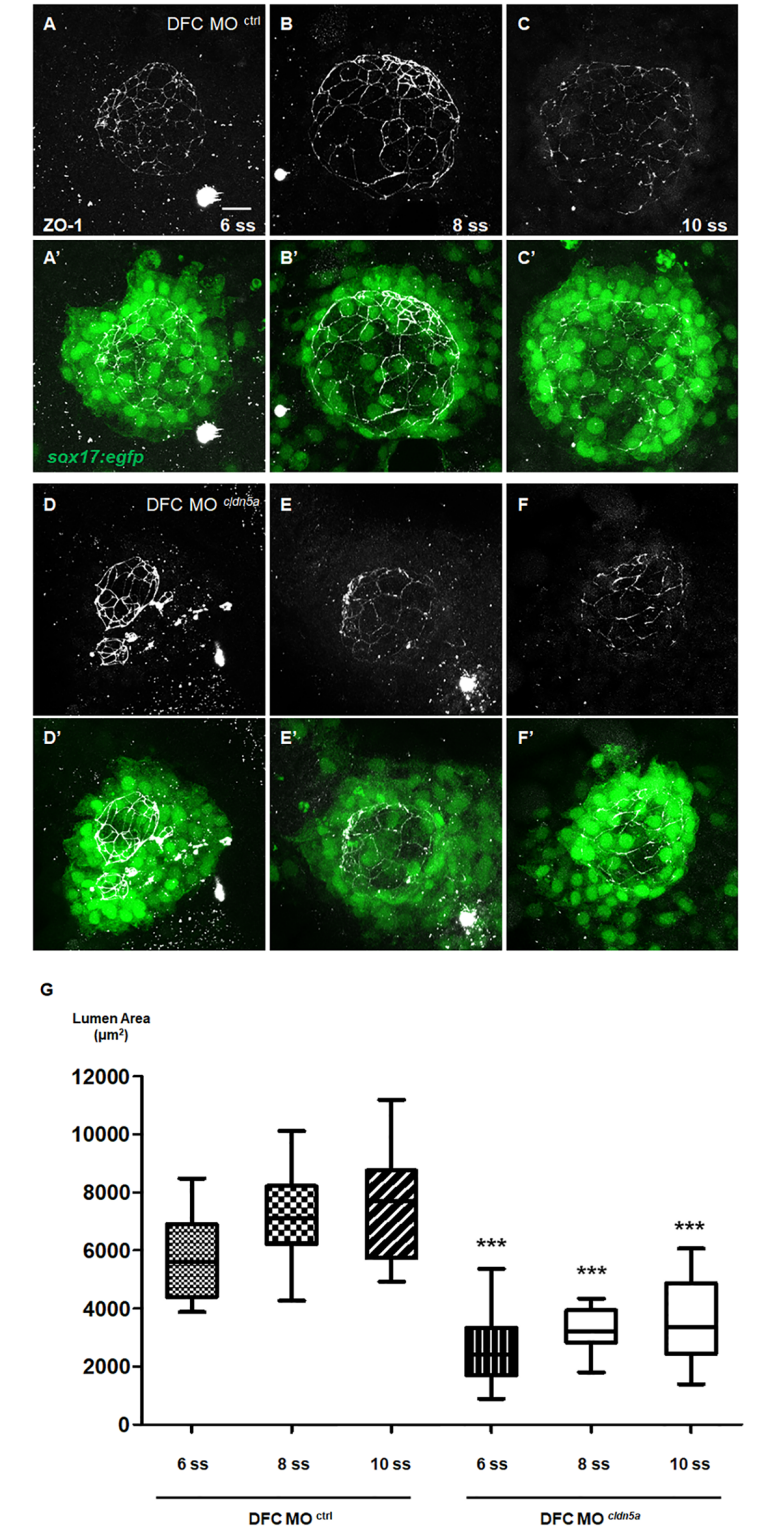
Downregulation of *cldn5a* resulted in defective KV lumen in zebrafish. **(A—F)** Maximum intensity projection images of ZO-1 (grey) and *sox17*:*egfp*-positive KV cells (green) in 6 ss, 8 ss and 10 ss embryos. Representative images of the DFC control morphants (A—C) and DFC *cldn5a* morphants (D—F). **(G)** Statistical box and whisker graph (DFC control morphants at 6 ss; n = 20, DFC control morphants at 8 ss; n = 31, DFC control morphants at 10 ss; n = 15, DFC *cldn5a* morphants at 6 ss; n = 28, DFC *cldn5a* morphants at 8 ss; n = 21, DFC *cldn5a* morphants at 10 ss; n = 18). Scale bar: 20 μm.

We observed that the KV lumen was small in DFC *cldn5a* morphants; accordingly, we investigated the role of *cldn5a* in KV lumen formation and expansion. First, *sox17* (a marker for DFC specification [50]) promoter-induced EGFP was stained at the 75% epiboly stage, when the DFCs collectively migrate towards the vegetal pole. In both DFC control and DFC *cldn5a* morphants, each DFC cluster was normally maintained without fragmentation (Fig 3A–3C), indicating that DFC specification was not affected by *cldn5a*. Next, we measured the KV lumen area over time. Fortunately, the localization of ZO-1 at the luminal surface of the KV lumen was not affected by the loss of *cldn5a* (S6 Fig). Thus, we measured the area enclosed by ZO-1 in Z-stack images by maximum intensity projection. However, since the morphology of the ZO-1-positive KV lumen in DFC *cldn5a* morphants was a distorted circle, we used the Closed Beizer tool in ZEN software which automatically calculates the enclosed area, rather than obtaining KV^max^, which multiplies the length of the longest radius. The KV lumen areas of DFC *cldn5a* morphants and DFC control morphants were 2599 ± 207.3 μm^2^ and 5798 ± 315.1 μm^2^ at 6 ss, 3286 ± 159.4 μm^2^ and 7157 ± 281.0 μm^2^ at 8 ss, and 3526 ± 348.9 μm^2^ and 7515 ± 446.0 μm^2^ at 10 ss, respectively (Fig 5). Considering the KV lumen area and the number of cells in the KV together (S5 Fig), these data suggest that the KV lumen was not fully inflated in DFC *cldn5a* morphants. In addition, we injected *mCherry* or *mCherry-cldn5a* mRNA with *cldn5a* morpholino to rescue the KV lumen area in *cldn5a* morphants. Compared with *cldn5a* morphants with *mCherry* (lumen area: 2183 ± 176.3 μm^2^), *cldn5a* morphants with *mCherry*-*cldn5a* exhibited recovery of the KV lumen area (4216 ± 274.2 μm^2^) (S7 Fig).

**Fig 6:**
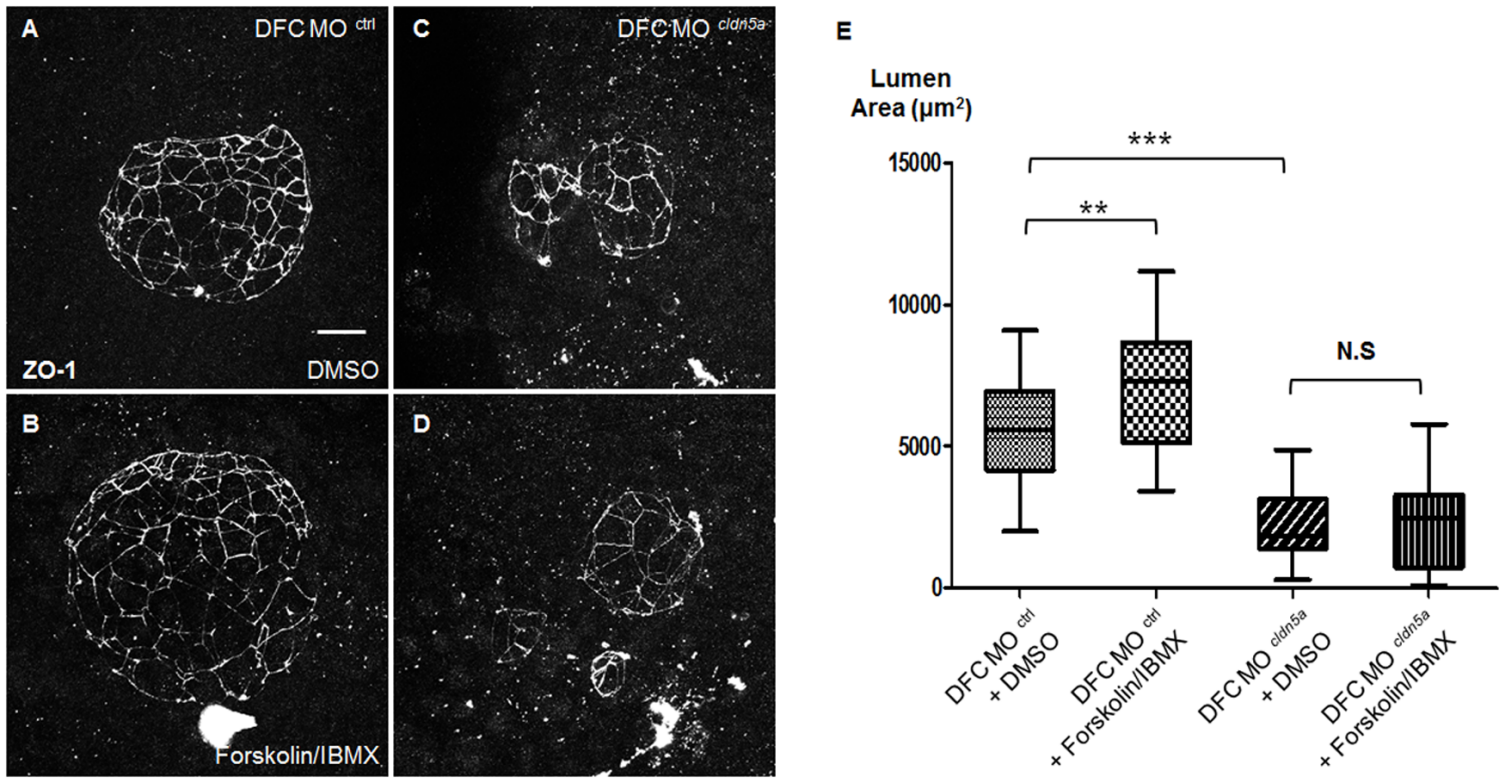
Treatment of forskolin and IBMX failed to recover the KV lumen size in DFC *cldn5a* morphants. **(A—D)** Maximum intensity projection images of ZO-1 in 6 ss embryos. Representative images of DFC control morphants treated with DMSO (A) or forskolin/IBMX (B). Representative images of DFC *cldn5a* morphants treated with DMSO (C) or forskolin/IBMX (D). **(E)** Statistical column bar graph (DFC control morphants with DMSO; n = 30, DFC control morphants with forskolin/IBMX; n = 28, DFC *cldn5a* morphants with DMSO; n = 19, DFC *cldn5a* morphants with forskolin/IBMX; n = 22). *** depicts *p* < 0.001, ** depicts *p* < 0.01, N.S. depicts *p* > 0.05. Error bars indicate s.e.m. Scale bar: 20 μm.

Claudins are major constituents of tight junction strands and previous papers have reported the size-selective loosening of blood-brain barrier in *cldn5* knockout mice and an under-inflated ventricular lumen in *cldn5a* morphant zebrafish [37, 42]. Thus, we assumed that paracellular permeability was increased and fluid influx probably leaked through the intercellular space between the KV epithelial cells. Thus, recovery of the malformed KV lumen size in DFC *cldn5a* morphants by increasing the fluid influx was verified. From 90% epiboly to 6 ss, zebrafish embryos were treated with a mixture of 10 μM forskolin and 40 μM IBMX which increases fluid secretion through Cftr by elevating the intracellular levels of cAMP by activating adenylyl cyclase and inhibiting phosphodiesterase activity, respectively [14, 18, 19]. Combined treatment with forskolin and IBMX successfully increased the KV lumen area in DFC control morphants (7020 ± 423.0 versus 5410 ± 307.9 μm^2^, Fig 6A, 6B and 6E). However, the combined treatment failed to increase the KV lumen area in DFC *cldn5a* morphants (2117 ± 275.0 versus 2266 ± 347.3 μm^2^, Fig 6C–6E), indicating that the reinforced fluid influx leaked through the intercellular space between weakly-adhered KV epithelial cells in the absence of *cldn5a*. Thus, we suggest that *cldn5a* is required for KV lumen inflation by supporting paracellular junctions within KV epithelial cells.

## Discussion

In the present study, we identified that *cldn5a* was highly expressed at the luminal surface of KV epithelial cells and sealed the paracellular spaces as a functional component of tight junction during KV lumen formation. DFC cluster, maintained by cadherin-based adherens junctions [12], actively protrudes towards the vegetal pole showing filopodia and adheres to the overlying enveloping layer until polarization and detachment from the enveloping layer to form the rosette structure [9, 11]. Then, the rosette structure is apically clustered and exhibits a centered lumen, called KV. Our results revealed that *cldn5a* was expressed from the bud stages and was abundantly localized at the luminal surface of KV when the DFC detached from the enveloping layer and underwent apical clustering and lumen formation (Fig 1). In addition, *sox17*:*egfp*-positive cells in DFC *cldn5a* morphants at 75% epiboly were grouped together without fragmentation and the number of *sox17*:*egfp*-positive cells at the bud stage was similar (S5 Fig). Thus, we assumed that *cldn5a* plays a role in sealing the KV luminal surface, but not in maintaining DFC cluster, nor in DFC-enveloping layer adhesion.

KV lumen is expanded by fluid influx through the Cftr channel, and the proper size of the KV lumen is important for left-right asymmetric development [14, 15, 18, 35]. It was reported that not only the reduced size of KV lumen, but also over-inflation of the KV lumen by reinforced activation of the Cftr channel disrupted regional cell shape changes of KV epithelial cells and caused defective left-right asymmetry [14, 18, 19, 35]. In this regard, *cldn5a* downregulation resulted in a decreased KV lumen area (Fig 5). Moreover, combined treatment with forskolin and IBMX, which increases the fluid influx through the Cftr channel, was ineffective to restore the KV lumen size in DFC *cldn5a* morphants (Fig 6). It suggests that the fluid leaked through the defective intercellular space between the KV epithelial cells in DFC *cldn5a* morphants, although a more direct way such as dextran injection into KV to probe the loss of paracellular tightness in DFC *cldn5a* morphants is needed. Nevertheless, the incomplete, but partially formed KV lumen in DFC *cldn5a* morphants still persisted in the absence of paracellular tightness. We suspect that regional cell shape changes by extracellular matrix accumulation and *rock2b*-mediated cytoskeletal rearrangement led to hollow spaces between the cells [19–21]. In addition, normal localization of ZO-1, the cytoplasmic adaptor protein for the tight junction component, at the luminal surface in DFC *cldn5a* morphants implies the presence of another tight junction protein, which sustains the partially formed lumen between the weakly adhered cells.

As a ciliated organ, the number of motile monocilia within the KV is important for the functional signal generation. More than 30 cilia are needed to drive sufficient force of fluid flow and induce the early left-right markers, *dand5* and *spaw* [45, 51]. In addition, the adequate length of cilia is important for left-right asymmetric development, as longer or shorter cilia lengths disrupt organ laterality by regulating the cilia beating frequency [27, 45, 52]. Although ciliogenesis occurs by complicated mechanisms, including FGF, Hedgehog, Notch, and nc-Wnt signaling pathways [53], Navis et al. [14] suggested that the generation of cilia is not related to lumen expansion. In their model, *cftr* mutant, cilia were generated within the center of the KV lumen even though the luminal space was undetectable [14]. In our case, a decreased number, but normal length of cilia was located near the under-inflated lumen in DFC *cldn5a* morphants (Fig 4). This discrepancy could be interpreted as ciliogenesis is related to apical-basal polarity, rather than sufficient space. Considering that neighboring KV epithelial cells lose their paracellular tightness, we suspect that a part of non-lumen-facing KV cells might lose their apical-basal polarity in DFC *cldn5a* morphants. However, the relevance between lumen formation and the potency of ciliogenesis needs further elucidation.

As a consequence of the reduced number of cilia within the defective KV lumen, *dand5* was aberrantly expressed in DFC *cldn5a* morphants (Fig 3), indicating KV dysfunction. Besides, the expression of *dand5* was decreased in DFC *cldn5a* morphants (Fig 3J), though cilia-driven fluid dynamics were inversely related to *dand5* expression [45]. We suspect that this might be the combined result of defective KV with high permeability and a decreased number of cilia. One possible explanation is that the initial transcription of *dand5* might be reduced due to the reduction of KV lumen-facing cells. Another one is that leaking fluid might affect the degradation of *dand5*. In any case, the expression of *spaw* was not fully restricted to the left LPM showing bilateral or right-sided patterns, as a consequence of the aberrant expression of *dand5*, and the heart laterality was disrupted in DFC *cldn5a* morphants (Figs 2 and 3).

The current study utilized morpholino-based approach to determine the role of *cldn5a* in zebrafish. Two types of *cldn5a* morphants showed similar phenotypes of disrupted heart laterality and exogenous RNA rescued the *cldn5a* morpholino-induced phenotypes. Several recent papers reported that genome-engineered mutants failed to recapitulate the morpholino-induced phenotypes [54–56]. Although genetic compensation might be induced in mutant embryos [57], morpholino-based studies should be carefully considered whether their phenotypes are driven by off-target effects. In this regard, we conducted CRISPR/Cas9-mediated *cldn5a* mutation and analyzed whether heart laterality might be affected in *cldn5a* crispants [58]. First, we constructed two types of *cldn5a*-targeting guide RNA (gRNA) chimera which directs Cas9 to forward and reverse strands of the genomic *cldn5a*, respectively. gRNA2, which targets reverse strand, successfully mutated *cldn5a*. Moreover, gRNA2-mediated *cldn5a* crispants showed high rate (61%) of disrupted heart laterality (S8 Fig). Thus, together with the morpholino-based studies, these data further support the role of *cldn5a* in organ laterality determination.

Taken together, we identified the functional tight junction component, *cldn5a*, in KV, and its role in proper inflation of KV lumen and left-right asymmetric organ development. Thus, these results advance our understanding of KV lumen formation and organ laterality.

## Supporting information

S1 File Supporting methods. (DOCX)

S1 Fig Expression patterns and immunoreactivity of recombinant mCherry, mCherry-Cldn5a and mCherry-cldn5b. **(A—C**) Expression patterns of recombinant proteins in 6 hpf embryo. Representative images of recombinant mCherry (A), mCherry-Cldn5a (B) and mCherry-Cldn5b (C). **(A’–C’)** Immunoreactivity to anti-mammal Cldn5 antibody of recombinant proteins. Representative images of recombinant mCherry (A’), mCherry-Cldn5a (B’) and mCherry-Cldn5b (C’). Scale bar: 20 μm.
(TIF)

S2 Fig Expression of Cldn5 in KV was ablated by two types of *cldn5a* translation-blocking MO. **(A—F)** Maximum intensity projection images of Cldn5 (red) and *sox17*:*egfp*-positive KV cells (green) in 6 ss embryos. Representative images of standard control MO injected embryo (n = 9) (A), *cldn5a* translation-blocking MO^1^ injected embryo (n = 11) (B), *cldn5a* translation-blocking MO^2^ injected embryo (n = 11) (C), DFC-specific control morphants (n = 8) (D), DFC-specific *cldn5a* MO^1^ injected embryo (n = 6) (E), and DFC-specific *cldn5a* MO^2^ injected embryo (n = 7) (F). Scale bar: 20 μm.
(TIF)

S3 Fig Serial expression of *cldn5a* and *cldn5b*. Relative mRNA expression rate of *cldn5a* and *cldn5b* was normalized by *eef1a1l1* according to the developmental stages from shield (6 hpf) to prim-5 (24 hpf).
(TIF)

S4 Fig Gross morphology and heart laterality defects of *cldn5a* MO^1^ and MO^2^ injected embryos. **(A—C)** Gross morphology of control morphants (A), *cldn5a* MO^1^ injected embryo (B), and *cldn5a* MO^2^ injected embryo (C). **(D—F)** Visualization of heart by *in situ* hybridization of *cmlc1* in 30 hpf embryos. Representative images of control morphants (D), *cldn5a* MO^1^ injected embryo (E), and *cldn5a* MO^2^ injected embryo (F). **(E)** Statistical stacked bar graph (blue; normal, orange; middle, grey; reversed, control morphants; n = 68, *cldn5a* MO^1^ injected embryos; n = 55, and *cldn5a* MO^2^ injected embryos; n = 53).
(TIF)

S5 Fig Status of DFC cluster and KV consisting cell number in DFC *cldn5a* morphants. **(A—B)** Visualization of DFCs by immunostaining of *sox17*-promoter induced EGFP in 75% epiboly embryos. Representative images of DFC control morphants (A) and DFC *cldn5a* morphants (B). **(C)** Statistical stacked bar graph (blue; normal, orange; fragmented, DFC control morphants; n = 22, DFC *cldn5a* morphants; n = 38). **(D—M)** Maximum intensity projection images of *sox17*:*egfp*-positive KV lineage cells in DFC control and *cldn5a* morphants from bud to 10 ss. (D—H) Representative images of the DFC control morphants. (I—M) Representative images of the DFC *cldn5a* morphants. **(N)** Statistical column bar graph (DFC control morphants at bud; n = 18, DFC *cldn5a* morphants at bud; n = 18, DFC control morphants at 3 ss; n = 25, DFC *cldn5a* morphants at 3 ss; n = 30, DFC control morphants at 6 ss; n = 23, DFC *cldn5a* morphants at 6 ss; n = 31, DFC control morphants at 8 ss; n = 19, DFC *cldn5a* morphants at 8 ss; n = 20, DFC control morphants at 10 ss; n = 16, DFC *cldn5a* morphants at 10 ss; n = 19). *** depicts *p* < 0.001, ** depicts *p* < 0.01, N.S. depicts *p* > 0.05. Error bars indicate s.e.m. Scale bar: 20 μm.
(TIF)

S6 Fig Localization of ZO-1 was not altered in DFC *cldn5a* morphants. **(A—F)** Single plane images of ZO-1 (grey) and *sox17*:*egfp*-positive KV cells (green) in 6 ss and 8 ss embryos. Representative images of DFC *cldn5a* morphants at 6 ss (A—C) and 8 ss (D—F). Scale bar: 20 μm.
(TIF)

S7 Fig KV lumen area of *cldn5a* morphants was restored by exogenous *cldn5a* mRNA. **(A—C)** Maximum intensity projection images of ZO-1 in 6 ss embryos. Representative images of control morphants with *mCherry* (A), *cldn5a* morphants with *mCherry* (B), and *cldn5a* morphants with *mCherry-cldn5a* (C). **(D)** Statistical box and whisker graph (control morphants with *mCherry*; n = 25, *cldn5a* morphants with *mCherry*; n = 39, *cldn5a* morphants with *mCherry-cldn5a*; n = 43) *** depicts *p* < 0.001. Error bars indicates s.e.m. Scale bar: 20 μm.
(TIF)

S8 Fig Laterality of heart was disrupted in *cldn5a* crispants. **(A)** Partial nucleotide sequences of *cldn5a* coding sequence (1–150 among 648) and two types of *cldn5a* targeting gRNA sequences. **(B)** Representative images of WT-like, type1 and type2 embryos at 30 hpf. **(C)** Stacked bar graph (blue; WT-like, orange; type1, grey; type2, WT; n = 24, 40 pg of gRNA1 injected embryos; n = 45, 40 pg of gRNA2 injected embryos; n = 41, 80 pg of *cas9* mRNA injected embryos; n = 52, 40 pg of gRNA1 and 80 pg of *cas9* mRNA injected embryos; n = 55, 40 pg of gRNA2 and 80 pg of *cas9* mRNA injected embryos; n = 42). **(D—F)** Visualization of a heart by *in situ* hybridization of *cmlc1* in 30 hpf embryos. Representative images of WT (F), gRNA1 crispants (G), and gRNA2 crispants (H). **(G)** Stacked bar graph (blue; normal, orange; middle, grey; reversed, WT; n = 24, only gRNA1 injected embryos; n = 43, only gRNA2 injected embryos; n = 40, only *cas9* mRNA injected embryos; n = 52, gRNA1 crispants; n = 28, gRNA2 crispants; n = 36). **(H)** T7E1 analysis of *cldn5a* crispants. **(I)** Representative mutations of *cldn5a* gene in gRNA2 crispants.
(TIF)
