# Supplementary material for: Claudin5a is required for proper inflation of Kupffer's vesicle lumen and organ laterality
Source: PLoS One. 2017 Aug 3;12(8):e0182047. doi: 10.1371/journal.pone.0182047 (PMC5542556; doi:10.1371/journal.pone.0182047)
Supplement: S1 File — (DOCX) [file pone.0182047.s001.docx]

**Real-time polymerase chain reaction (qPCR)**

Primer sequences for PCR amplification are as below : *cldn5a* forward (5’-CTC TGG AAG CTC CGG GTA CT-3’) and reverse (5’-CCT CTT GTC GTA TTC CCC GT-3’), *cldn5b* forward (5’-CAT CGT CGC AGG CTT GTT TG-3’) and reverse (5’-TGG CGT AGG GAA CTT GAG GA-3’), *eef1a1l1* forward (5’-CTG GAG GCC AGC TCA AAC AT-3’) and reverse (5’-ATC AAG AAG AGT AGT ACC GCT AGC ATT AC-3’).

**Whole mount immunohistochemistry**

*Tg(sox17:egfp*) embryos were fixed with 4% PFA for overnight at 4℃ and dehydrated with methanol for long storage at –20℃. Then the embryos were treated with collagenase Ⅰ for 10 min at room temperature. After the collagenase Ⅰ treatment, the embryos were transferred to the blocking solution (5% bovine serum albumin, 10% normal goat serum in PBST) and incubated for 3 hours at room temperature. Blocking solution was replaced with mouse anti-GFP IgG (1:200, Invitrogen) containing solution and the embryos were incubated at 4℃ overnight. A series of washing steps with PBDTT were performed and the embryos were treated with biotin-labeled goat anti-mouse IgG (1:1000, Vector Laboratories) in blocking solution for overnight at 4℃. Then, the embryos were washed with PBDTT several times and treated with AB mixture (PK-6100, Vector Laboratories) for 1 hour and transferred into 3,3'-diaminobenzidine (DAB) solution (Sigma-Aldrich). Color reaction was performed by adding 0.3% H_2_O_2_ into DAB solution where the embryos were immersed. Stained embryos were mounted in glycerol and photographed with AxioCam ICC-1 camera on Zeiss Stemi 2000.

**Genome editing using CRISPR/Cas9 system**

From cloning gRNA vector constructs to *in vitro* transcription, detailed protocol was following the method as described in the previous report [58]. For generation of *cldn5a*-targeting gRNA constructs, two pairs of oligonucleotides, gRNA1 forward (5’-TAG GTC TGA TCC TGT GCG TCT G-3’) and reverse (5’-AAA CCA GAC GCA CAG GAT CAG A-3’), and gRNA2 forward (5’-TAG GTC TGC GCG ACC ACG ATG T-3’) and reverse (5’-AAA CAC ATC GTG GTC GCG CAG A-3’), were annealed and incorporated into BsmBⅠ-digested pT7-gRNA (Addgene plasmid #46759), respectively.

**Genomic DNA isolation**

To isolate genomic DNA (gDNA), embryos were incubated with SNET buffer (20 mM Tris-HCl, 5 mM EDTA, 400 mM NaCl, 1% sodium dodecyl sulfate, 400 μg/mL proteinase K, pH 8.0) at 55℃ for overnight. Then, an equal volume of PCI solution (phenol: chloroform: isoamyl alcohol) was added to the gDNA containing SNET buffer, and the organic and aqueous phases were separated by centrifugation of the samples at 666 g for 5 min. Supernatant was transferred to the new tube, and the gDNA was precipitated by adding an equal volume of isopropanol. After collecting the precipitated gDNA by centrifugation at 13,250 g for 15 min at 4℃, carefully removed the isopropanol and rinsed the pellet of gDNA with 1 mL of 70% ethanol. Then, the pellet was dried for 20 min, and gDNA pellet was dissolved in nuclease free water.

**T7E1 assay**

Wild-type (WT) and *cldn5a* crispant gDNA amplicons were melted and reannealed to hybridize DNA strands randomly. Then, the gDNA amplicons were incubated with T7E1 restriction enzyme for 15 min at 37℃.
