# Supplementary figures and images for: Claudin5a is required for proper inflation of Kupffer's vesicle lumen and organ laterality
Source: PLoS One. 2017 Aug 3;12(8):e0182047. doi: 10.1371/journal.pone.0182047 (PMC5542556; doi:10.1371/journal.pone.0182047)

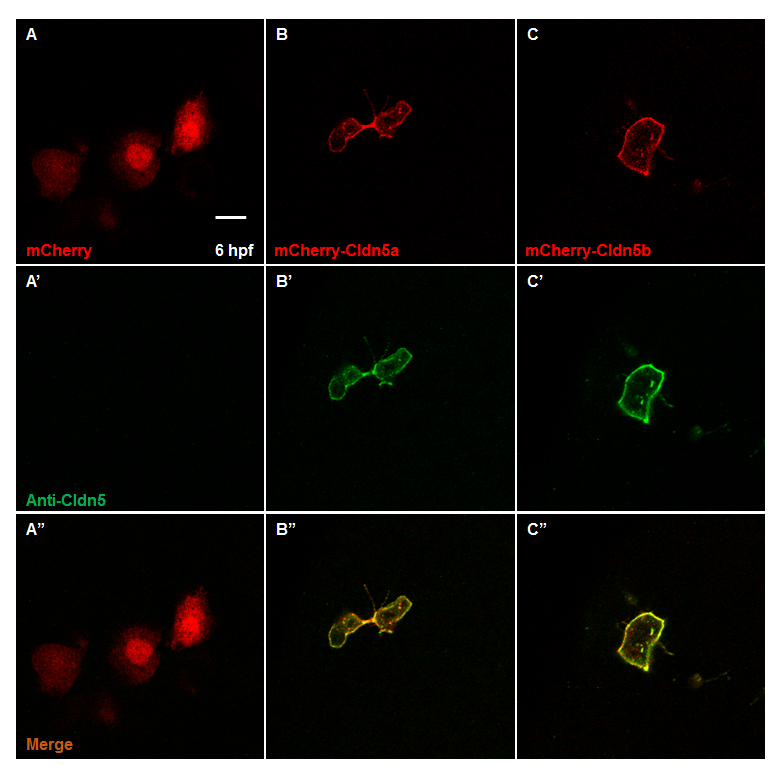

Supplement: S1 Fig — (A—C) Expression patterns of recombinant proteins in 6 hpf embryo. Representative images of recombinant mCherry (A), mCherry-Cldn5a (B) and mCherry-Cldn5b (C). (A’–C’) Immunoreactivity to anti-mammal Cldn5 antibody of recombinant proteins. Representative images of recombinant mCherry (A’), mCherry-Cldn5a (B’) and mCherry-Cldn5b (C’). Scale bar: 20 μm. (TIF) [file pone.0182047.s002.tif]

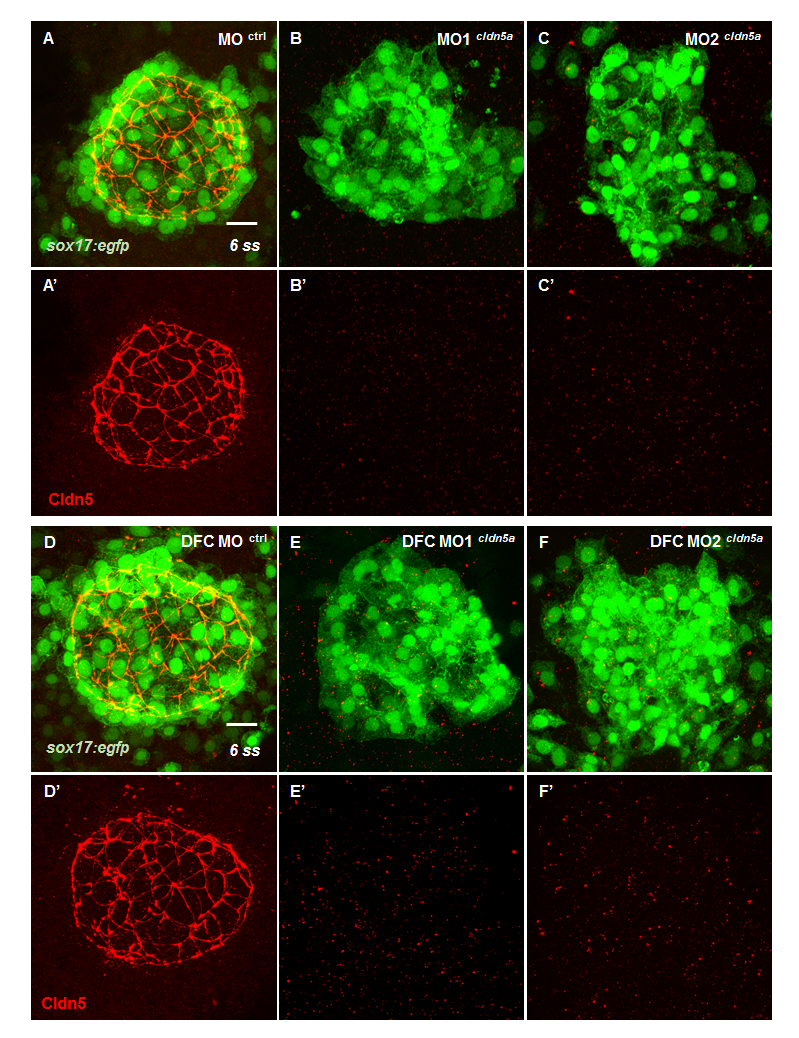

Supplement: S2 Fig — (A—F) Maximum intensity projection images of Cldn5 (red) and sox17:egfp-positive KV cells (green) in 6 ss embryos. Representative images of standard control MO injected embryo (n = 9) (A), cldn5a translation-blocking MO1 injected embryo (n = 11) (B), cldn5a translation-blocking MO2 injected embryo (n = 11) (C), DFC-specific control morphants (n = 8) (D), DFC-specific cldn5a MO1 injected embryo (n = 6) (E), and DFC-specific cldn5a MO2 injected embryo (n = 7) (F). Scale bar: 20 μm. (TIF) [file pone.0182047.s003.tif]

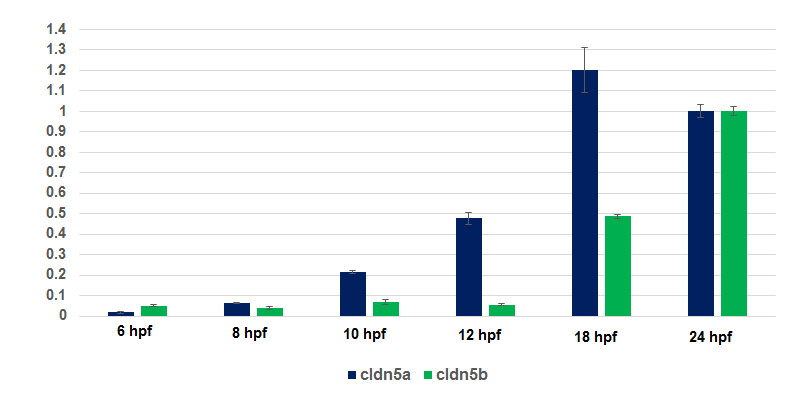

Supplement: S3 Fig — Relative mRNA expression rate of cldn5a and cldn5b was normalized by eef1a1l1 according to the developmental stages from shield (6 hpf) to prim-5 (24 hpf). (TIF) [file pone.0182047.s004.tif]

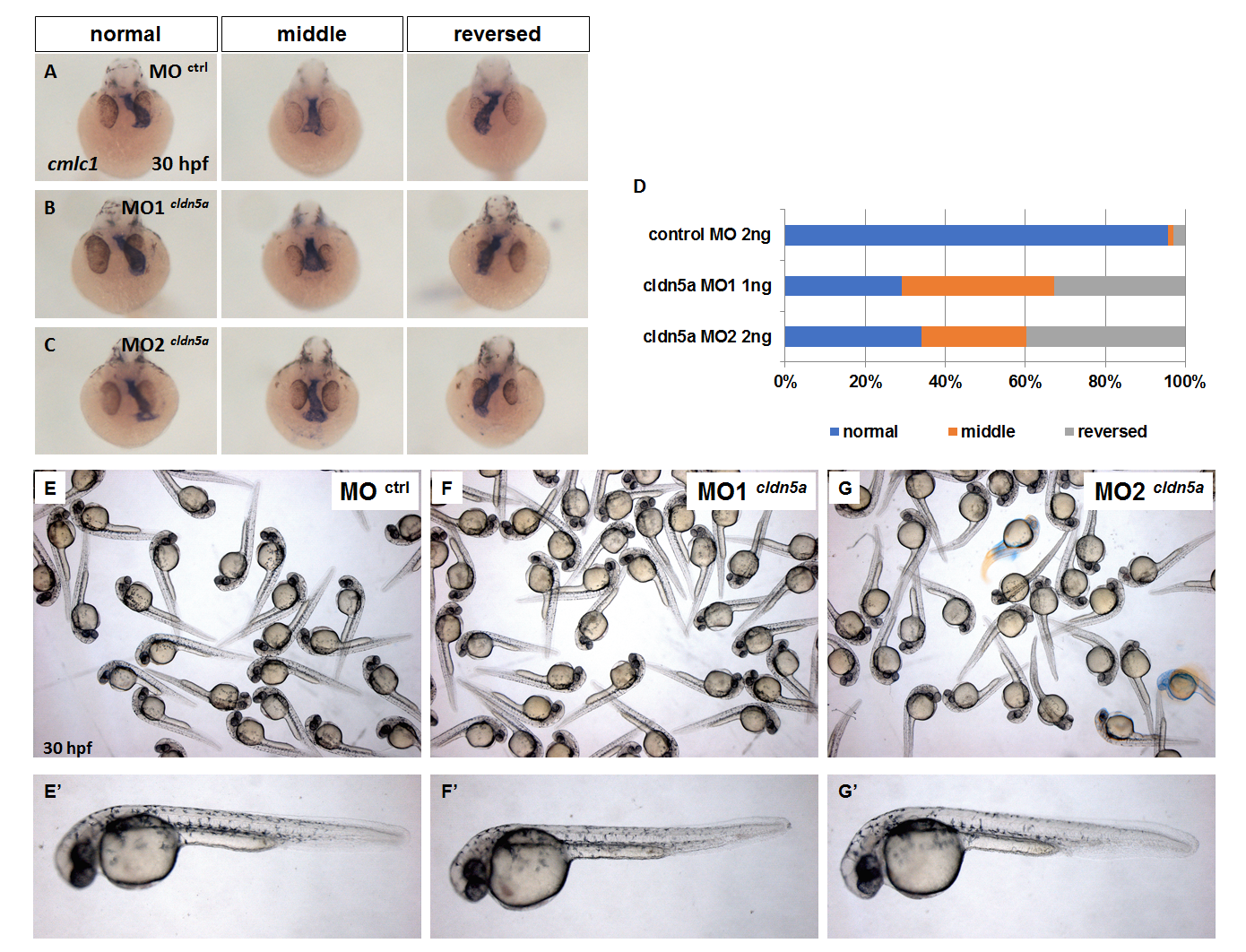

Supplement: S4 Fig — (A—C) Gross morphology of control morphants (A), cldn5a MO1 injected embryo (B), and cldn5a MO2 injected embryo (C). (D—F) Visualization of heart by in situ hybridization of cmlc1 in 30 hpf embryos. Representative images of control morphants (D), cldn5a MO1 injected embryo (E), and cldn5a MO2 injected embryo (F). (E) Statistical stacked bar graph (blue; normal, orange; middle, grey; reversed, control morphants; n = 68, cldn5a MO1 injected embryos; n = 55, and cldn5a MO2 injected embryos; n = 53). (TIF) [file pone.0182047.s005.tif]

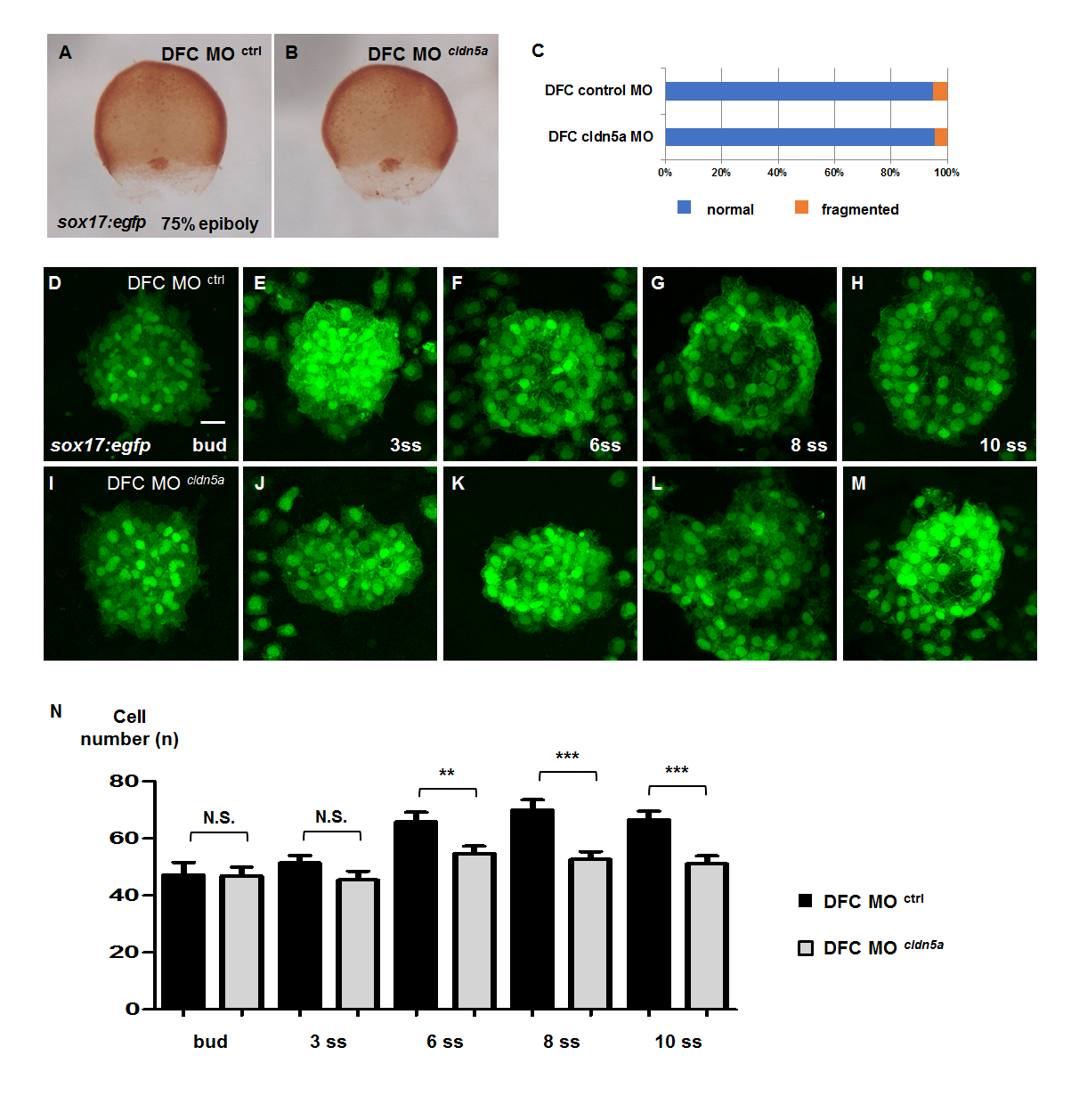

Supplement: S5 Fig — (A—B) Visualization of DFCs by immunostaining of sox17-promoter induced EGFP in 75% epiboly embryos. Representative images of DFC control morphants (A) and DFC cldn5a morphants (B). (C) Statistical stacked bar graph (blue; normal, orange; fragmented, DFC control morphants; n = 22, DFC cldn5a morphants; n = 38). (D—M) Maximum intensity projection images of sox17:egfp-positive KV lineage cells in DFC control and cldn5a morphants from bud to 10 ss. (D—H) Representative images of the DFC control morphants. (I—M) Representative images of the DFC cldn5a morphants. (N) Statistical column bar graph (DFC control morphants at bud; n = 18, DFC cldn5a morphants at bud; n = 18, DFC control morphants at 3 ss; n = 25, DFC cldn5a morphants at 3 ss; n = 30, DFC control morphants at 6 ss; n = 23, DFC cldn5a morphants at 6 ss; n = 31, DFC control morphants at 8 ss; n = 19, DFC cldn5a morphants at 8 ss; n = 20, DFC control morphants at 10 ss; n = 16, DFC cldn5a morphants at 10 ss; n = 19). *** depicts p < 0.001, ** depicts p < 0.01, N.S. depicts p > 0.05. Error bars indicate s.e.m. Scale bar: 20 μm. (TIF) [file pone.0182047.s006.tif]

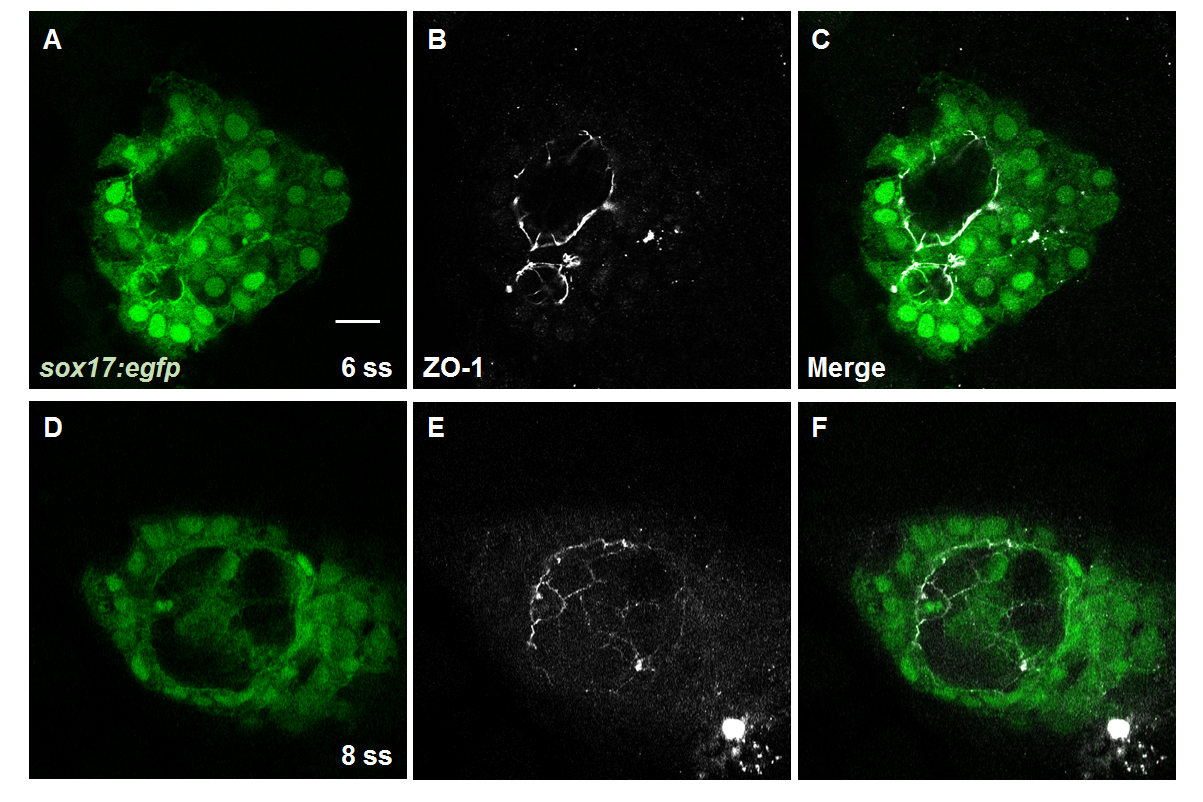

Supplement: S6 Fig — (A—F) Single plane images of ZO-1 (grey) and sox17:egfp-positive KV cells (green) in 6 ss and 8 ss embryos. Representative images of DFC cldn5a morphants at 6 ss (A—C) and 8 ss (D—F). Scale bar: 20 μm. (TIF) [file pone.0182047.s007.tif]

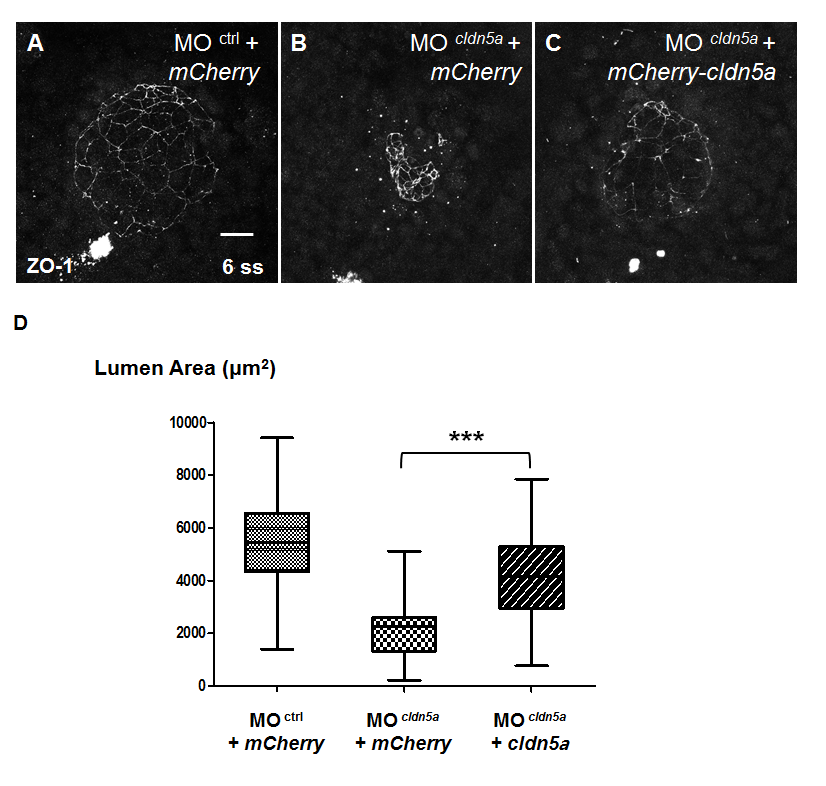

Supplement: S7 Fig — (A—C) Maximum intensity projection images of ZO-1 in 6 ss embryos. Representative images of control morphants with mCherry (A), cldn5a morphants with mCherry (B), and cldn5a morphants with mCherry-cldn5a (C). (D) Statistical box and whisker graph (control morphants with mCherry; n = 25, cldn5a morphants with mCherry; n = 39, cldn5a morphants with mCherry-cldn5a; n = 43) *** depicts p < 0.001. Error bars indicates s.e.m. Scale bar: 20 μm. (TIF) [file pone.0182047.s008.tif]

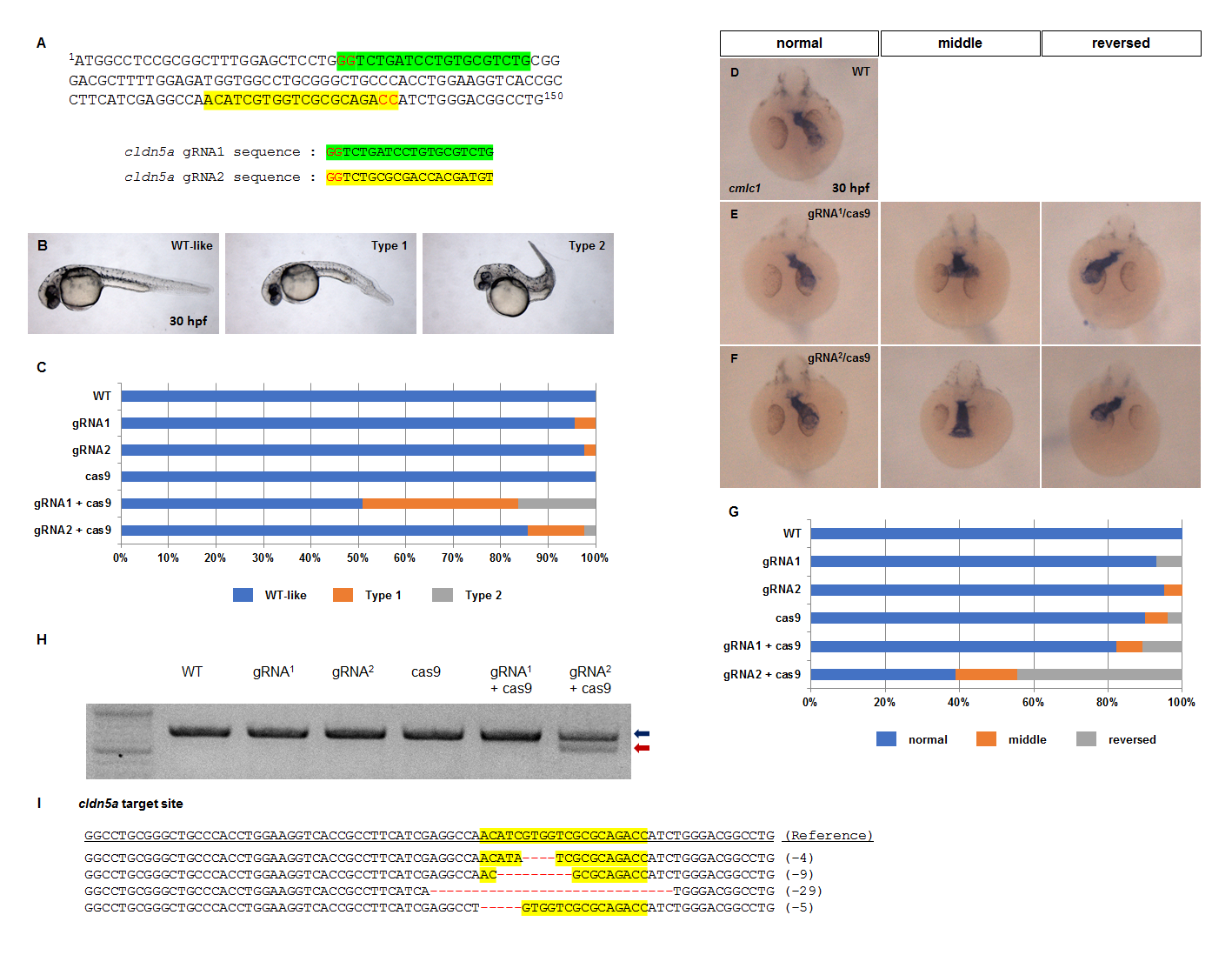

Supplement: S8 Fig — (A) Partial nucleotide sequences of cldn5a coding sequence (1–150 among 648) and two types of cldn5a targeting gRNA sequences. (B) Representative images of WT-like, type1 and type2 embryos at 30 hpf. (C) Stacked bar graph (blue; WT-like, orange; type1, grey; type2, WT; n = 24, 40 pg of gRNA1 injected embryos; n = 45, 40 pg of gRNA2 injected embryos; n = 41, 80 pg of cas9 mRNA injected embryos; n = 52, 40 pg of gRNA1 and 80 pg of cas9 mRNA injected embryos; n = 55, 40 pg of gRNA2 and 80 pg of cas9 mRNA injected embryos; n = 42). (D—F) Visualization of a heart by in situ hybridization of cmlc1 in 30 hpf embryos. Representative images of WT (F), gRNA1 crispants (G), and gRNA2 crispants (H). (G) Stacked bar graph (blue; normal, orange; middle, grey; reversed, WT; n = 24, only gRNA1 injected embryos; n = 43, only gRNA2 injected embryos; n = 40, only cas9 mRNA injected embryos; n = 52, gRNA1 crispants; n = 28, gRNA2 crispants; n = 36). (H) T7E1 analysis of cldn5a crispants. (I) Representative mutations of cldn5a gene in gRNA2 crispants. (TIF) [file pone.0182047.s009.tif]
